# Factors Associated with Mental Illness in a Primary Healthcare Setting in the Kingdom of Saudi Arabia: A Case–Control Study

**DOI:** 10.3390/healthcare12131298

**Published:** 2024-06-28

**Authors:** Abdulaziz S. Alangari, Faris Fatani, Nouf Binhowaimel, Hanan M. Al Kadri, Awad Alshahrani, Badr F. Al Khateeb, Aljohrah I. Aldubikhi, Mona I. Bin Amer, Afrah Alsaif, Ashraf El-Metwally

**Affiliations:** 1 College of Public Health and Health Informatics, King Saud bin Abdulaziz University for Health Sciences, Riyadh 11481, Saudi Arabia; 2 King Abdullah International Medical Research Center, Riyadh 11481, Saudi Arabia; 3 Riyadh Second Health Cluster, Riyadh 11525, Saudi Arabia; 4 Department of Obstetrics and Gynecology, King Abdulaziz Medical City, Ministry of the National Guard-Health Affairs, Riyadh 11426, Saudi Arabia; 5 Department of Medicine, King Abdulaziz Medical City, Ministry of the National Guard-Health Affairs, Riyadh 11426, Saudi Arabia; 6 College of Medicine, King Saud bin Abdulaziz University for Health Sciences, Riyadh 11481, Saudi Arabia; 7 Department of Family Medicine, King Abdulaziz Medical City, Ministry of the National Guard-Health Affairs, Riyadh 11426, Saudi Arabia; 8 College of Health Sciences, Saudi Electronic University, Riyadh 13316, Saudi Arabia; 9 College of Health Information Systems, Inaya Medical Colleges, Riyadh 13541, Saudi Arabia

**Keywords:** mental illness, primary care, Saudi Arabia, stroke, diabetes, obesity

## Abstract

Background/Objectives: Mental health conditions are a leading cause of morbidity and mortality worldwide, with a 13% rise within the last decade. This study aimed to investigate the factors associated with mental illness in patients presenting to a primary healthcare center in the Kingdom of Saudi Arabia (KSA). Methods: Data were extracted from the electronic health records of 46 primary care centers in Riyadh, Saudi Arabia from March 2022 to March 2023. A total of 2418 age-matched mental health cases and controls were evaluated. Descriptive and logistic regression analyses were performed to examine the distribution and association of relevant risk factors. A total of 1209 cases were age-matched to 1209 controls presenting to a primary clinic. Results: The odds of mental illness in females were 2.2 times that of males. Clinical conditions associated with mental illness were stroke, diabetes, and obesity. Neurodegenerative disorders were also associated with mental illness. Conclusions: Primary clinics in the KSA can serve as focal points in removing the barriers to mental healthcare. There is an urgent need to spread awareness, remove the stigma, and provide appropriate care and referrals for mental health conditions in the KSA.

## 1. Introduction

Mental health conditions are a leading cause of morbidity and mortality worldwide, with a 13% rise within the last decade [1]. In 2019, it was estimated that mental disorders attributed to around 125–418 million disability-adjusted life years (DALYs) [2,3]. According to the Saudi National Mental Health Survey (SNMHS), 34.2% of Saudis are diagnosed with a mental condition at some point in their lives [4]. The most common mental health disorders in the Kingdom of Saudi Arabia (KSA) are anxiety disorders, mood disorders, disruptive behavior disorders, eating disorders, and substance use disorders [5]. There are several barriers to timely and appropriate care for mental conditions due to stigma, lack of awareness, and lack of quality care [6,7]. Moreover, those who perceived a need for treatment indicated that they wanted to handle the problem on their own [8]. 

KSA is considered a high-income country, with a total population of 32.2 million people. The median age of the population is 29 years, and 63% of Saudis are under the age of 30. Males represent 61% (19.7 million) of the population, while females account for 39% (12.5 million) [9]. There are few studies on mental health conditions in the KSA that assess risk factors. A study conducted during the COVID-19 pandemic found that predictors of mental illness were female gender, younger age group, single/divorced marital status, lower educational level, lower socio-economic status, non-Saudi residential status, student status, small family size, and elderly house member status [10]. Studies on the student population found lack of physical activity and sleep to be the strongest predictors of poor mental health [11,12]. In addition, smoking, ill health, and low resilience have been predictive of mental health conditions in the KSA [10,13]. 

Often, manifestations of mental illness stem from a myriad of physical symptoms, also known as “somatic symptom disorders”, for which patients visit primary clinics on multiple occasions [14]. The “Collaborative Care” model integrates mental health treatment into primary care settings. The model improves access to mental health services, emphasizes evidence-based treatments, and ensures the continuous monitoring and adjustment of care plans to meet patients’ needs effectively. It has proven to be effective in alleviating depressive symptoms and healthcare costs and improving patient satisfaction and quality of life in high-income countries [15,16]. Studies have shown that the overall treatment for mental disorders has increased by 2.6 times in primary clinics and 2.2 times in psychiatric clinics [17]. Given the post-COVID-19 era, there is a greater urgency to promote the primary care sector for the diagnosis and treatment of mental health conditions [18]. 

Supporting the role of primary care physicians (PCPs) in the diagnosis, treatment, and appropriate referral of mental illnesses has widespread implications in alleviating the burden of poor mental health in the KSA. The newly proposed “Model of Care” works to reform the existing healthcare system by prioritizing non-communicable disease burdens in primary settings [19]. This is essential in addressing the mental health burden, as the KSA suffers from a severe shortage of psychiatrists. Given the dearth of research on the subject in this region, identifying mental health factors will help formulate diagnostics, plan treatment strategies, and promote awareness of mental health and well-being through PCPs. This study aimed to investigate the factors associated with mental illness in patients presenting to a primary healthcare center in the Kingdom of Saudi Arabia

## 2. Materials and Methods

### 2.1. Design and Study Population

This study used the electronic health records of the Second Health Cluster in Riyadh, Saudi Arabia. This cluster was established in 2018 and includes 46 primary care centers. The data were extracted retrospectively from March 2022 to March 2023. Cases included all those with any diagnosed mental illness based on asking the patient if they had been diagnosed with any mental disorder before by a licensed professional. Controls were those who did not report any mental health illness and were chosen randomly. Each patient with a mental health disease (case) was matched with one patient without a mental health disease (control) (1:1 basis). The matching process was conducted using age (exact years of age). Ethical approval for this study was approved by the IRB of King Fahad Medical City (IRB#: 22-397E). Informed consent was waived since the dataset included secondary data analysis and deidentified information of the participants.

### 2.2. Measures

The variables considered in this study were as follows: Age (≤25, 25–35, 36–45, 46–60, or ≥60), Sex (Male or Female), Body Mass Index (BMI), Diabetes (Yes or No), Hypertension (Yes or No), Asthma or Obstructive Pulmonary Disease (Yes or No), Stroke (Yes or No), Hyperlipidemia (Yes or No), and Dementia/Alzheimer’s/Parkinson’s Disease (Yes or No). The clinical assessment was performed by asking the patients if they had been diagnosed before or not.

### 2.3. Statistical Analysis

Descriptive statistics were presented as means, medians, and standard deviations (SD) for continuous data and frequencies with percentages for categorical data. Comparison between variables was conducted using the *t*-test, Mann–Whitney test, or χ^2^ test, as appropriate. A conditional logistic regression analysis was conducted to identify factors associated with the likelihood of being diagnosed with mental illness. All baseline clinical and demographic characteristics were considered in the analysis. Different factors that were found to be significant in univariate analysis were considered for inclusion in the final multivariable model. The strength of association was reported as an odds ratio (OR) with the Wald test *p*-value. All tests were two-sided and a *p*-value < 0.05 was considered significant. The statistical software Stata (version 11, StataCorp, College Station, TX, USA) was used to perform the analysis.

## 3. Results

A total number of 1209 patients with mental health diseases were identified and matched with a similar number of patients without mental health diseases. As shown in Table 1, age distribution was similar in the two groups: those in the middle age categories predominated and those in the young or older age categories had less representation. Moreover, sex distribution differed significantly between the two groups, with a higher proportion of females in the group with mental health diseases. Body mass index (BMI) was also significantly higher in those with mental diseases. Similar results were observed when comparing the distribution of diabetes (*p* < 0.0001), asthma (or other obstructive pulmonary diseases; *p* < 0.001), and stroke (*p* < 0.0001) across the two groups, with consistently significantly higher proportions observed in those with mental health diseases. However, the proportion of patients with hypertension (*p* = 0.089), dyslipidemia (*p* = 0.317), or dementia/Alzheimer’s/Parkinson’s disease (*p* = 0.056) did not differ between the two groups.

**Figure 1:**
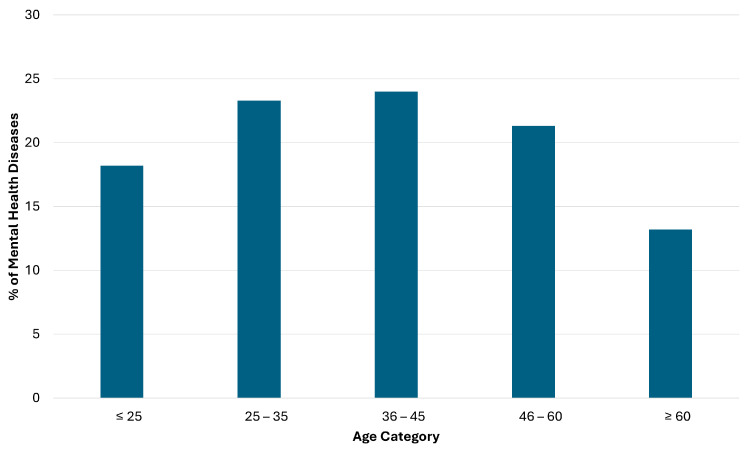
Mental health disease distribution by age category.

Figure 1 presents the distribution of mental health disease by age category. The figure suggests that mental health disease was disproportionately less prevalent in those in the youngest and oldest age groups, and manifested much more frequently in the middle age groups (those aged 24–35 or 36–45 years). In the conditional logistic regression analysis conducted to identify factors associated with the likelihood of being diagnosed with a mental illness, only sex, BMI, diabetes, stroke, and asthma/obstructive pulmonary disease variables were statistically significant in the univariate analysis (Table 2). However, this significant association was consistent in the final multivariable model, in which a significantly increased likelihood of mental health disease was observed in females (OR = 2.12, 95% CI: 1.77–2.55, *p* < 0.0001), patients with diabetes (OR = 1.98, 95% CI: 1.34–2.92, *p* = 0.001), and stroke (OR = 4.59, 95% CI: 2.07–10.19, *p* < 0.0001). Those with asthma/obstructive pulmonary disease had a significantly reduced likelihood of being diagnosed with a mental health disease (OR = 0.45, 95% CI: 0.25–0.79, *p* = 0.005). Further, increased BMI was associated with a marked significant increase in the likelihood of being diagnosed with a mental health disease (OR = 1.02, 95% CI: 1.01–1.04, *p* = 0.001).

## 4. Discussion

A novel, case–control, age-matched design within a primary care setting was used to identify factors associated with mental illness in the KSA. In total, 1209 cases were age-matched to 1209 controls presenting at primary healthcare centers in the country. Several important predictors of poor mental health were noted, with the greatest number of cases in two age categories: 25–35 and 36–45 years. According to the SNMHS, the age brackets with the highest percentage of mental illness are 15–24 years (40%) and 25–34 years (40%) [4]. The survey reports a rate of 29% in the 35–44 year bracket. Separately, the odds of mental illness in females were 2.2 times those of males. In a systematic review and meta-analysis of factors associated with mental health problems in the KSA (2019–2021), it was found that female sex was associated with poor mental health in over 90% of the studies reviewed [10]. Even in the pre-COVID-19 pandemic years, studies highlighted that women were more prone to suffering poor mental health compared to men [20,21]. It is known that women are more prone to worry, accompanied by a mounting level of fear and discomfort [20,22]. Biological differences, specifically menstrual patterns, affect mood and coping abilities in women [23,24]. Overall, genetic and environmental factors play a complex role in the differential development of mental health problems in men and women. 

Another important factor to be considered in mental health gender differences is that men and women experience different types of mental health problems. While women exhibit internalizing behaviors (anxiety, depression, phobias), men present with more externalizing conditions (drug abuse and explosive disorder). The SNMHS recorded separation anxiety disorder (13%), major depressive disorder (8.9%), social phobia (7%), and attention deficit disorder (6%) as most common in Saudi females, while separation anxiety disorder (11%), attention deficit disorder (10%), social phobia (4.3%), and bipolar disorder (4%) were most frequent in men [4]. Further, the survey revealed that substance abuse had a 2.4% prevalence in females and a 2.9% prevalence in males. It is possible, nevertheless, that reporting bias is present in studies of mental health disorders, as there is a huge stigma attached to such conditions in the KSA and other countries. A study reported that men are less likely to pursue treatment and share their problems, which suggests a significant risk of underdiagnosis for both internalizing and externalizing disorders in men [25].

The results are consistent with previous studies showing a strong association between poor mental health and obesity. A large review of studies reported statistically significant odds ratios for developing depression in obese patients (CI of OR: 1.21–5.8) and developing obesity in depressed patients (CI of OR: 1.18–3.76), with a stronger association observed in women [26]. The study showed a 2% increase in the odds of mental disease in those with increased BMI scores (*p* = 0.001). This has huge implications, both for research and clinical purposes. Globally, rates of mental illness and obesity are growing both independently and in conjunction. It is imperative to understand the impact of obesity on the prevalence of mental illness in the KSA, particularly in the younger age brackets, which had higher frequencies of obesity in the current study’s cohort. This finding suggests the critical role of the primary care practitioner in the early identification and management of obesity for timely mental health prevention and promotion. 

Similarly, diabetic patients often develop mental health illnesses, and those with poor mental health develop diabetes [27,28]. Subsequently, those suffering from diabetes have been reported to have a 2- to 3-fold increase in the odds of being diagnosed with a mental illness [29]. The current study contributes important evidence from the KSA and demonstrates the odds of developing diabetes in those with a mental illness to be approximately 2 times higher compared to those without a mental illness (CI: 1.34–2.92, *p* = 0.001). The relationship between diabetes and mental health can be completely independent, such that the worsening or improvement of one disease does not affect the other. It is possible, however, that diabetes gives rise to psychiatric disorders such as psychotic disorders (including schizophrenia) or vice-versa in a complex pathobiological mechanism [27]. Further, it is possible that the clinical presentation of common diabetic events (hypoglycemic and ketoacidosis) manifests as mental conditions such as panic attacks [30]. Additionally, pharmaco-epidemiologic data show that some psychiatric medications such as antipsychotics produce diabetes as a side effect [27,31]. Of grave concern is the fact that close to 50% of mental illnesses go undiagnosed in patients with diabetes [32]. Again, this has huge implications for PCPs, who are often the focal point in diagnosis and treatment initiation. Our study lends important data for the support of PCPs in the extended management of mental illnesses and their complex overlap with obesity and diabetes. 

Studies based on strokes from the KSA are very limited; the incidence rates for the first incidence of strokes in the KSA have been reported to be 29.8 per 100,000 people per year [33,34]. A recent study on coping mechanisms in patients who had suffered a stroke found that most of the participants had mild or moderate depression, while more than 30% had mild to severe anxiety [35]. From 43 stroke patients in the current sample, 35 screened positive for a mental health illness. The results also show that the odds of poor mental health in persons suffering from strokes are about 4.59 times those of people who have not suffered from a stroke (*p* < 0.0001). These results are consistent with previous studies. The pathophysiology of depression following a stroke is multifactorial; it is imperative that neurologists, mental health practitioners, and PCPs work together to address this challenge through appropriate treatment strategies. 

Interestingly, the current study found that among those suffering from asthma, the odds of mental illness were reduced by 55% (CI: 0.25–0.79) compared to those not suffering from asthma. Results from the World Mental Health Survey [36] found that anxiety disorders were associated with asthma in adults (pooled OR = 1.3–1.8). Some countries, such as Colombia, reported ORs of less than one, as in the current study. The authors from the World Mental Health Survey suggest that this may be due to the limited number of cases in each survey; this study had a total of 69 patients with asthma. It is possible that the OR was below one due to the limited sample size and statistical loss of power. It is also possible that those suffering from asthma did not have poor mental health due to the constant care provided to them for their physical condition. Furthermore, no association was found between metabolic disorders (dyslipidemia) and neurodegeneration (dementia, Alzheimer’s, Parkinson’s). Even though neurodegeneration is associated with depressive symptoms [37], this was not observed, probably due to a low number of cases (10) in this cohort. Similarly, only four patients with dyslipidemia were present. 

Primary clinics in Saudi Arabia can serve as focal points in removing barriers to mental healthcare by offering accessible, integrated, and holistic services that address both physical and mental health needs. These clinics are widely distributed, making them accessible to diverse populations, including those in rural areas. By incorporating mental health screenings into routine care, they enable early detection and intervention, reducing the progression of mental illnesses. Additionally, the normalization of mental healthcare in these settings helps reduce stigma, while long-term patient–provider relationships support continuous monitoring and care. Primary clinics can also provide education, resources, and strong referral networks to specialized services, ensuring comprehensive and coordinated care. By advocating for supportive policies and engaging with the community, primary clinics can enhance awareness and foster a more supportive environment for mental healthcare.

### 4.1. Strengths

The strengths of this study lie in its sample size, primary care setting, and case–control design. There are very few epidemiological studies from this region of the world and even fewer from primary healthcare clinics. A notable strength of this study is its reflection of a real primary care setting scenario, providing valuable insights into the factors associated with mental illness as they naturally occur in everyday clinical practice. By conducting the research in a primary care environment, this study captured a diverse patient population with a wide range of medical conditions and socio-demographic backgrounds, thereby enhancing the generalizability of the findings. This real-world context ensures that the results apply to typical primary care settings, making the conclusions and recommendations highly relevant for primary care providers seeking to identify and address mental health issues among their patients. There is a growing burden of mental health illness in the KSA, and there is an urgent need to address this. Due to the extreme shortage of well-trained mental healthcare workers in the region, the current study presents a vital role for the primary care physician in mental illness diagnosis and management.

### 4.2. Limitations

This study has several important limitations. Firstly, the reliance on self-reported data introduced the possibility of reporting bias, whereby participants may have underreported or overreported symptoms of mental illness due to stigma, recall inaccuracies, or social desirability. Secondly, this study did not employ standardized screening tools to assess mental health conditions, which could have led to variability in the identification and classification of mental health issues, affecting the validity of the findings. Additionally, as all mental health illnesses were grouped together, it was not clear which physical conditions were associated with which mental illnesses. Given the limited studies from the KSA, it would be beneficial to unlock the associations between specific mental health conditions. Thirdly, the duration of the medical illness was not considered in the analysis, which could be a significant factor influencing mental health outcomes. This omission limits the ability to fully understand the relationship between the chronicity of medical conditions and the development of mental health disorders. Lastly, we had very few cases of neurodegenerative disorders and metabolic disorders (such as dyslipidemia); hence, no statistically valid inference could be made. These limitations suggest that the findings should be interpreted with caution, and further research using more rigorous methodologies is recommended.

## 5. Conclusions

This study’s results show that among sociodemographic factors, female gender was strongly associated with the odds of having a mental illness. From the clinical factors, obesity, diabetes, and strokes were significantly associated with mental illness. The current study provides strong support for the diagnosis and management of mental illnesses in the general population through appropriate care in the primary care setting. In addition, primary clinics in the KSA are able to serve as satellites in spreading awareness, removing stigma, and providing appropriate care and referral for mental health conditions.

## Figures and Tables

**Table 1:** Demographic and clinical characteristics of cases (n = 1209) and controls (n = 1209).

Characteristic	No Mental Health Disease (Controls)	Mental Health Disease (Cases)	*p*
	n (%)	n (%)	
**Age Category (years)**			1.0
≤25	220 (18.2)	220 (18.2)	
25–35	282 (23.3)	282 (23.3)	
36–45	290 (24.0)	290 (24.0)	
46–60	258 (21.3)	258 (21.3)	
≥60	159 (13.2)	159 (13.2)	
**Sex**			**<0.0001**
Male	620 (51.3)	404 (33.4)	
Female	589 (48.7)	805 (66.6)	
**Body Mass Index (BMI)**			
Mean (SD)	28 (6.2)	29.1 (6.9)	**<0.0001**
Median (IQR)	27.3 (23.6–31.6)	28.7 (24.0–34.0)	**<0.0001**
Minimum	15.1	15.1	
Maximum	49.9	49.8	
**Diabetes**			**<0.0001**
No	1160 (95.9)	1104 (91.3)	
Yes	49 (4.1)	105 (8.7)	
**Hypertension**			0.089
No	1181 (97.7)	1167 (96.5)	
Yes	28 (2.3)	42 (3.5)	
**Asthma/Obstructive Pulmonary Disease**			**0.001**
No	1188 (89.2)	1161 (96.0)	
Yes	21 (1.7)	48 (4)	
**Stroke**			**<0.0001**
No	1201 (99.3)	1174 (97.1)	
Yes	8 (0.7)	35 (2.9)	
**Hyperlipidemia**			0.317
No	1206 (99.8)	1208 (99.9)	
Yes	3 (0.2)	1 (0.1)	
**Dementia/Alzheimer’s/Parkinson’s Disease**			0.056
No	1207 (99.8)	1201 (99.3)	
Yes	2 (0.2)	8 (0.7)	

*p*: *p*-value; χ^2^ test for categorical data, *t*-test for comparison of means, and Mann–Whitney test for comparison of medians. Bold *p*-values denote statistical significance at the *p* < 0.05 level.

**Table 2:** Conditional logistic regression for variables associated with mental health diseases.

Characteristic	Univariate Analysis		Multivariable Analysis	
	Odds Ratio (95% CI)	*p*	Odds Ratio (95% CI)	*p*
**Sex**		**<0.0001**		**<0.0001**
Female	2.21 (1.85–2.64)		2.12 (1.77–2.55)	
Male	1		1	
**Body Mass Index (BMI)**	1.03 (1.02–1.05)	**<0.0001**	1.02 (1.01–1.04)	**0.001**
**Diabetes**		**<0.0001**		**0.001**
Yes	2.40 (1.66–3.47)		1.98 (1.34–2.92)	
No	1		1	
**Stroke**		**<0.0001**		**<0.0001**
Yes	4.38 (2.03–9.43)		4.59 (2.07–10.19)	
No	1		1	
**Asthma/Obstructive Pulmonary Disease**		**0.001**		**0.005**
Yes	0.40 (0.23–0.69)		0.45 (0.25–0.79)	
No	1		1	

Bold *p*-values denote statistical significance at the *p* < 0.05 level.

## Data Availability

The data that support the findings of this study are available from the Saudi Ministry of Health. Restrictions apply to the availability of these data, which were used under license for this study. Data are available from the authors with the permission of the Saudi Ministry of Health.
